# Transcriptomic Analysis of *Rhodococcus opacus* R7 Grown on *o*-Xylene by RNA-Seq

**DOI:** 10.3389/fmicb.2020.01808

**Published:** 2020-08-12

**Authors:** Jessica Zampolli, Alessandra Di Canito, Andrea Manconi, Luciano Milanesi, Patrizia Di Gennaro, Alessandro Orro

**Affiliations:** ^1^Department of Biotechnology and Biosciences, University of Milano-Bicocca, Milan, Italy; ^2^Institute of Biomedical Technologies, National Research Council, CNR, Milan, Italy

**Keywords:** *Rhodococcus opacus*, *o*-xylene, RNA-seq, oxygenases, stress response, environmental contamination

## Abstract

Xylenes are considered one of the most common hazardous sources of environmental contamination. The biodegradation of these compounds has been often reported, rarer the ability to oxidize the *ortho*-isomer. Among few *o*-xylene-degrading bacteria, *Rhodococcus opacus* R7 is well known for its capability to degrade diverse aromatic hydrocarbons and toxic compounds, including *o*-xylene as only carbon and energy source. This work shows for the first time the RNA-seq approach to elucidate the genetic determinants involved in the *o*-xylene degradation pathway in *R. opacus* R7. Transcriptomic data showed 542 differentially expressed genes that are associated with the oxidation of aromatic hydrocarbons and stress response, osmotic regulation and central metabolism. Gene ontology (GO) enrichment and KEGG pathway analysis confirmed significant changes in aromatic compound catabolic processes, fatty acid metabolism, *beta*-oxidation, TCA cycle enzymes, and biosynthesis of metabolites when cells are cultured in the presence of *o*-xylene. Interestingly, the most up-regulated genes belong to the *akb* gene cluster encoding for the ethylbenzene (Akb) dioxygenase system. Moreover, the transcriptomic approach allowed identifying candidate enzymes involved in R7 *o*-xylene degradation for their likely participation in the formation of the metabolites that have been previously identified. Overall, this approach supports the identification of several oxidative systems likely involved in *o*-xylene metabolism confirming that *R. opacus* R7 possesses a redundancy of sequences that converge in *o*-xylene degradation through R7 peculiar degradation pathway. This work advances our understanding of *o*-xylene metabolism in bacteria belonging to *Rhodococcus* genus and provides a framework of useful enzymes (molecular tools) that can be fruitfully targeted for optimized *o*-xylene consumption.

## Introduction

Xylenes are a group of synthetic aromatic hydrocarbons in which the position of methyl groups varies on the benzene ring. Worldwide, they are the second most important aromatic products for consumption in chemical manufacture (Industry Research, 2020). In the next 5 years, the xylene market is expected to further grow at an average rate of 3–4% per year (IHS Markit, 2017). Xylenes are intentionally or unintentionally released during storage and transportation and they are considered among the most common hazardous sources of environmental contamination.

Bacteria capable of degrading *m*-xylene or *p*-xylene under aerobic conditions are more common (Galli et al., 1992; Barbieri et al., 1993), while *ortho*-isomer degraders are less frequently reported due to its high recalcitrance. Among hydrocarbon-degrading bacteria, members of *Rhodococcus* genus represent a reservoir of intriguing genomic traits as well as functional diversity (Alvarez, 2019). Their importance is due to their metabolic and genetic flexibility and their tolerance to various stresses. Indeed, rhodococci are Gram-positive bacteria able to catabolize a remarkably wide range of organic and toxic compounds including *o*-xylene (Zampolli et al., 2019).

The metabolically versatile *Rhodococcus opacus* R7, isolated from a polycyclic aromatic hydrocarbon (PAH) contaminated site, is well-known for its ability to grow on naphthalene, several medium- and long-chain *n*-alkanes, and aromatic hydrocarbons belonging to BTEX group (benzene, toluene, ethylbenzene, and xylenes). Interestingly, R7 is able to grow on *o*-xylene as sole carbon and energy source (Di Gennaro et al., 2001, 2010; Di Canito et al., 2018). *R. opacus* R7 whole-genome analysis revealed numerous functions conferring the ability to degrade a large set of aliphatic, aromatic and PAHs distributed on the chromosome and five plasmids (pPDG1, pPDG2, pPDG3, pPDG4, and pPDG5) which explains the considerable catabolic pathway redundancy (Di Gennaro et al., 2014; Orro et al., 2015). Moreover, R7 has metabolic and genetic traits that grant this bacterium to thrive in harsh environments and in different stress conditions such as osmotic and oxidative stress, the presence of antibiotics, metals and other toxic compounds (Cappelletti et al., 2016). Besides, R7 strain was previously reported to be able to degrade *o*-xylene by a dioxygenase system (Akb) leading to the production of the corresponding dihydrodiol. On the other hand, the previous identification of main intermediates [2,3-dimethylphenol (2,3-DMP) and 3,4-dimethylphenol (3,4-DMP)] suggested that *o*-xylene degradation could proceed through a dioxygenation or two successive monooxygenations. Therefore, the identification of different genes encoding for several monooxygenases/phenol hydroxylases suggested the involvement of other oxygenases in the *o*-xylene degradation pathway in R7 strain (Di Canito et al., 2018).

The only other *Rhodococcus* strain reported to be able to perform *o*-xylene ring-oxidation through an oxygenase system is *Rhodococcus* sp. strain DK17 (Kim et al., 2004). The DK17 *o*-xylene oxygenase is described to perform a ring-oxidation leading to DMP that could be attributed to the dehydration of dihydrodiol deriving from the dioxygenation activity and subsequent formation of 3,4-dimethylcathechol (3,4-DMC) either by one dioxygenation or two monooxygenations (Kim et al., 2002). Thus, a broader approach to deeply investigate *o*-xylene degradation in *Rhodococcus* members is necessary to understand which genes and molecular mechanisms could be involved in this metabolism.

In this context, expression studies were exploited in *Rhodococcus* strains to establish aromatic hydrocarbon degradation pathways (Gonçalves et al., 2006; Patrauchan et al., 2005). The fast advancement of -*omic* technologies has facilitated the transition from the microarray approach toward the genome-wide gene expression techniques, such as RNA-seq (Costa et al., 2010).

To our knowledge, the only transcriptomic study that evaluated the expression profiles in the presence of BTEX compounds was conducted on *Pseudomonas putida* KT2440 using a DNA array technology. KT2440 TOL pathway was induced in the presence of the metabolizable toluene (and 3-methylbenzoate (3-MB) used as a specific positive inducer) or in the presence of the non-biodegradable *o*-xylene. In concomitance, these compounds generated a general stress response at several levels, reflected by the induction of genes known to respond to membrane damage, oxidative stress, and misfolding of soluble proteins principally caused by the solvent entrance (Domínguez-Cuevas et al., 2006).

Therefore, a broader understanding of the genes activated during *o*-xylene metabolism is necessary, especially regarding Gram-positive strains, including the resistant and versatile bacteria belonging to *Rhodococcus* genus.

This work reports for the first time the RNA-seq approach for the elucidation of the genetic determinants involved in the *o*-xylene degradation pathway in *R. opacus* R7. The interpretation of the functions and the related metabolic pathways of differentially expressed genes was investigated by Gene ontology (GO) and Kyoto Encyclopedia of Genes and Genomes (KEGG) enrichment analysis. The RNA-seq approach allowed to reconstruct a framework of functions converging in the *o*-xylene biodegradation.

## Materials and Methods

### Bacterial Strain and Growth Conditions

*Rhodococcus opacus* strain R7 (deposited to the Institute Pasteur Collection, CIP identification number 107348) used in this study was cultivated in M9 mineral medium supplemented with 20 mM malate. The overnight culture was washed in mineral medium and inoculated in fresh M9 mineral medium supplemented with *o*-xylene (1 g L^−1^) as only carbon and energy source. *o*-xylene was administrated into M9 mineral medium in an atmosphere saturated with the same aromatic compound in a sealed system. The reference condition was established inoculating R7 overnight culture in mineral medium with 20 mM malate after washing in the mineral medium. All cultures were incubated at 30°C under shaking (120 rpm) and they were harvested at the mid-exponential growth phase (optical density at 600 nm of 0.6 ± 0.05).

### RNA Extraction

Total RNA was extracted from 100 mL cultures of *R. opacus* R7 grown at 30°C on M9 mineral medium supplemented with *o*-xylene or malate. RNA extraction was obtained from each growth condition in triplicate and the protocol was performed using the RNA-Total RNA Mobio Isolation Kit (Qiagen Italia, Italy) according to the manufacturer’s instructions and at the end the DNase treatment was performed (provided with the kit). RNA quality was measured using Bioanalyzer Agilent 2100 supported with RNA 6000 Pico Agilent chip (Agilent, Italy). RNA quality check showed an integrity number (RNI value) major of 8 for both RNA samples. RNA concentration was evaluated with 6000 Nano kit (Agilent, Italy).

### RNA Sequencing

The RNA sequencing was performed with the Illumina platform (San Diego, CA, United States) after preparation of the sequencing library. Generated raw data are about 25.9 million pairs of reads (forward and reverse strands) for malate samples and 31.2 million for *o*-xylene samples. Quality of raw sequencing data was checked with FastQC (version 0.11.5). Then, sequencing reads were trimmed using Trim Galore (version 0.4.4) (Krueger, 2015), while duplicates were identified and removed using GPU-Dup Removal (Manconi et al., 2016). After data pre-processing FastQC reported an acceptable level of quality with a median over 30 and a vast majority of 100 nt-long sequences. After quality control, about one million of reads were depleted for both samples. The sequences of RNA-seq are submitted to ENA with the following accession number: PRJEB38098.

### RNA-Seq Data Analysis

A reference-based strategy was used to assemble the transcriptome. *R. opacus* R7 genome sequences constituted by one chromosome and five plasmids (pPDG1, pPDG2, pPDG3, pPDG4, and pPDG5) obtained in a previous work (Di Gennaro et al., 2014) was used as a reference for the RNA assembly. RNA-seq reads were mapped to the reference with TopHat aligner (Kim et al., 2013). RNA reads were processed with the cufflinks default pipeline (Trapnell et al., 2010) that assembles the reads of the two samples into a set of transcript fragments separately. The assembling process produced a total of 7299 and 7811 transcripts for malate and *o*-xylene samples with overall read mapping rate of 88.9% and 59.6%, respectively.

Finally, they were merged in a format suitable for the next differential gene expression analysis.

A total of 9664 open reading frames (ORFs) were predicted with Glimmer (Delcher et al., 2007) and annotated with RAST (Rapid Annotation using Subsystem Technology) (Aziz et al., 2008) in order to obtain functions and subsystems. About 6289 ORFs were functionally annotated grouped in 742 subsystems that represent biological functional roles (subsystems).

### RNA-Seq Differential Gene Expression

After quality control of RNA reads (deriving from malate and *o*-xylene growth conditions), they were analyzed with Cuffdiff software (Trapnell et al., 2013) with default parameters in order to obtain a set of loci with statistically different expression levels. Results were reported in a table showing loci information (identifier, scaffold, start and stop location), the expression values (proportional to the number of reads mapping the locus) and the significance level (*p*-value) (Supplementary Table S1). Finally, the loci were ranked and filtered by *p*-value (< 0.05) and annotated by assigning each locus the annotation from the corresponding ORF of the genomic DNA.

### Gene Cluster Annotation

The differentially regulated genes were manually analyzed by BLAST (Altschul et al., 1990) with *e*-value < 10 and CLUSTAL Omega (Thompson et al., 1994) alignments against reference sequences from the Uniprot database (The UniProt Consortium, 2019) and literature searches. Data resources used in gene cluster annotation include: KEGG (Kanehisa et al., 2017), Brenda (Schomburg et al., 2002), NCBI CDS (Marchler-Bauer et al., 2016) and Pfam (Bateman et al., 2004).

For each KEGG (Kanehisa et al., 2017) and GO (Mi et al., 2019) term the count of genes has been computed and compared between the two conditions in order to highlight the most relevant classifications.

### Quantitative Real-Time RT-PCR (RT-qPCR)

Reverse transcription was performed with iScriptcDNA Synthesis kit (BIO-RAD, Italy) to obtain the corresponding cDNAs. Total RNA was reverse-transcribed to obtain 200 ng cDNA. The reverse transcription was performed after 5 min at 25°C of denaturation followed of 1 h at 42°C and then 5 min of elongation at 85°C.

In order to verify the transcriptional induction of selected genes involved in *o*-xylene degradative pathways, Reverse Transcription (RT-) PCR experiments were performed by amplification of cDNA samples using the StepOnePlus Real-Time PCR System (Applied Biosystem, Italy). Amplification of *akbA1a*, *padAa, akbC*, *catA*, *czcO6*, *pcaH*, and 16S rRNA genes was performed in 10-μl qPCR volume contained 4.4 μl of the reverse-transcribed RNA samples, 5 μl of PowerUp SYBR Green Master Mix (Applied Biosystem, Thermo Fisher Scientific, Italy), and 300 nM of each primer listed in Table 1. Thermocycling conditions were as follows: 30 s at 95°C, followed by 40 cycles of 5 s at 95°C, 10 s at 60°C and 45 s at 72°C and one cycle of 15 s at 95°C, 1 min at 60°C and 15 s at 60°C. Expression of the housekeeping gene, 16S rRNA, was used as reference gene to normalize tested genes in *R*. *opacus* R7. The ΔΔCt method with 16S rRNA (Su et al., 2016) as reference gene was used to determine relative abundance of target transcripts in respect to malate. Data are expressed as mean ± standard deviation derived from at least three independent experiments.

**TABLE 1:** List of oligonucleotides used for RT-qPCR analyses.

**Oligonucleotide name**	**Sequence (5′ – 3′)**
RT-16S-R7f	TCGTGAGATGTTGGGTTAAG
RT-16S-R7r	CCTCTGTACCGGCCATTGTAG
RT-AkbA1-f	ATATGATCTTGGACAATGAGG
RT-AkbA1-r	ATTCTCCATATCAATCTCGGG
RT-padA-f	TGCCTGCACCGGGGTATGCAG
RT-padA-r	CCTTCTTCCTGAAACCGGCCT
RT-akbC-f	TCTACGGGCCGCAGATCGACA
RT-akbC-r	ATCAGCCGATAGAAGCGGGT
RT-catA-f	TGATGCCGCAACGGCCGGA
RT-catA-r	AGTCCGGGATGTTCGGGTGGA
RT-pcaH-f	TGTCCTTCAACGTCATCGACGGA
RT-pcaH-r	CCGCTCTCGTCGGCGAACA
RT-czcO6-f	GTCCCCAAGAAGCCGGAGTTCGA
RT-czcO6-r	CTTGGCTTCCTTGGCGATCTCG

In order to exclude DNA contamination, negative controls were performed by omitting the reverse transcriptase in RT-PCR experiments, which were conducted with the same temperature program and the same primer sets for 35 cycles of amplification.

## Results

### Overview of the Transcriptomic Analysis of *R. opacus* R7 During Growth on *o*-Xylene

The whole-transcriptomic analysis was performed to evaluate the gene expression of *Rhodococcus opacus* strain R7 cultivated in the presence of the most recalcitrant compound among BTEX compounds, *o*-xylene. This condition was compared to R7 growth in the presence of malate. The total RNA was extracted from *R*. *opacus* R7 cells (taken at the exponential growth phase) and it was sequenced by RNA-seq technology. The RNA assembling process produced a total of 7299 and 7811 transcripts for malate and *o*-xylene conditions with an overall read mapping rate of 88.9 and 59.6%, respectively. After quality control, a total of 30.7 and 25.4 million Illumina reads were detected in the presence of *o*-xylene and malate, respectively. The Illumina reads were used to calculate the differential gene expression [log_2_(fold change), from now also named as fold-change] under the different growth conditions.

**FIGURE 1:**
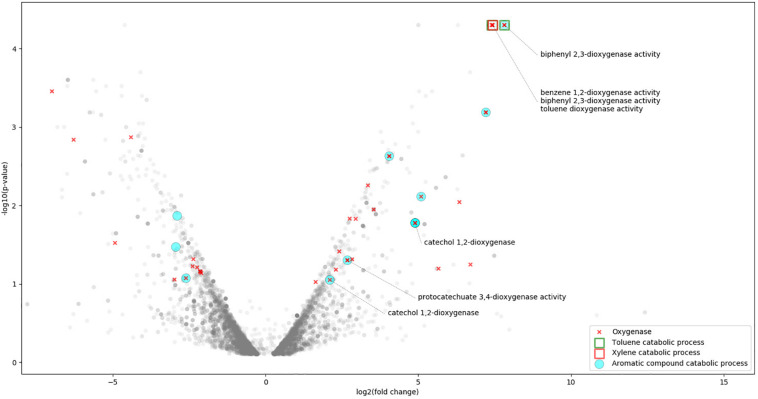
Expression profile of *R*. *opacus* R7 grown on *o*-xylene respect to malate condition in a V-plot with annotation based on GO analysis. V-plot shows *R*. *opacus* R7 statistically significant genes (*p*-value < 0.05) in the higher part of the plot (low *p*-value in the *y* axis); the right and the left parts of the plot (*x* axis) represent the DEGs under *o*-xylene or malate condition, respectively. The following GO categories are in the V-plot: Aromatic compound catabolic process, Toluene catabolic process, Xylene catabolic process, Toluene dioxygenase activity, Benzene 1,2-dioxygenase activity, Biphenyl 2,3-dioxygenase activity, Protocatechuate 3,4-dioxygenase activity, Catechol 1,2-dioxygenase, and Oxygenase.

All transcripts were functionally annotated with RAST (Aziz et al., 2008) after Glimmer prediction (Delcher et al., 2007) resulting in 9664 ORFs grouped in 742 subsystems. Among the total CDSs of the R7 genome, 94% were not showing appreciable changes under both conditions. Indeed, 9122 genes were not significantly affected by the substrates, while 542 were differentially expressed, considering fold change > 2 and *p*-value < 0.05. Among the differentially expressed genes (DEGs), 271 genes had higher expression levels (up-regulated), while the other 271 genes had lower expression levels (down-regulated) in the presence of *o*-xylene. The up-regulated genes showed a fold-change varying from 2 to 7.8 and the down-regulated genes from -2 to -7 (Figure 1 and Supplementary Table S1). Among the total DEGs, 43 DEGs showed abnormal fold-change values, of which 27 were up-regulated genes and 16 were down-regulated genes. The abnormal fold-change values, due to the expression values equal to zero in one of the two conditions (switch-off/-on effect), were corrected with a min-value approximation. Nevertheless, among all these 43 DEGs, 21 were preliminary annotated as hypothetical proteins (Supplementary Table S1).

According to the size, the R7 chromosome showed the highest number of up-regulated and down-regulated genes, 200 and 221 CDSs, respectively. However, the highest percentage of up-regulated genes relative to the total number of CDSs (55 and 10%) was present on pPDG5 plasmids and pPDG2 plasmids, respectively. While the highest percentage of down-regulated genes was on pPDG3 plasmid and it was around 4%.

**FIGURE 2:**
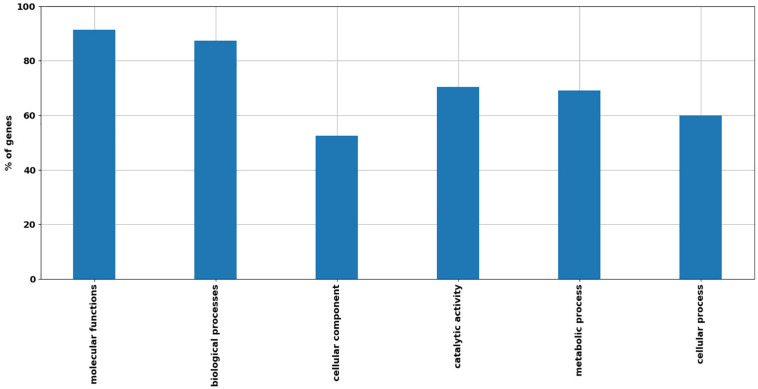
Distribution of main GO categories with respect to *R. opacus* R7 response to *o*-xylene and malate. Barplot shows the percentage of DEGs grouped in relevant GO categories, including the following categories: “Molecular functions,” “Biological processes,” “Cellular components,” “Catalytic activity,” “Metabolic processes,” and “Cellular processes.”

Open reading frames classified by RAST annotation were further annotated with GO using the Blast2Go platform (Mi et al., 2019) and KEGG (Kanehisa et al., 2017). Out of 502 assigned DEGs to GO categories, 91% were assigned to “molecular functions,” 87% to “biological processes,” and 53% to “cellular components.” High numbers of transcripts were classified under the GO categories viz “catalytic activity” 354 counts on 502 (70% of DEGs with GO annotation), “metabolic processes” 347 counts on 502 (69%), and “cellular processes” 301 counts on 502 (60%) (Figure 2).

**FIGURE 3:**
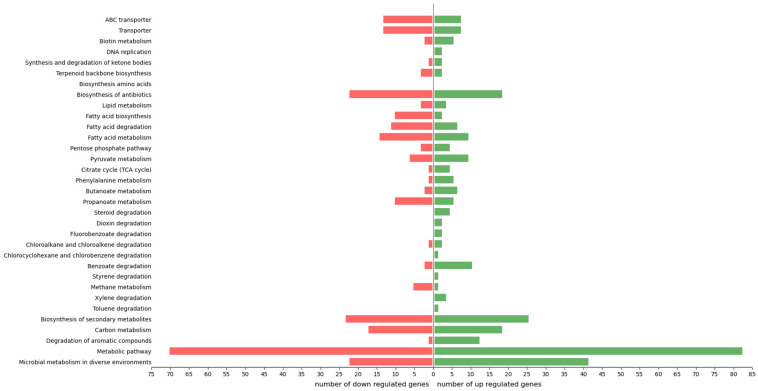
Distribution of *R. opacus* R7 responses to *o*-xylene and malate according to physiological categories. Barplot shows the number of up- and down-regulated genes grouped in relevant KEGG categories, such as microbial metabolism in diverse environments, metabolic pathway, degradation of aromatic compounds, carbon metabolism, biosynthesis of secondary metabolites, toluene degradation, xylene degradation, methane metabolism, styrene degradation, benzoate degradation, chlorocyclohexane and chlorobenzene degradation, chloroalkane and chloroalkene degradation, fluorobenzoate degradation, dioxin degradation, steroid degradation, propanoate metabolism, butanoate metabolism, phenylalanine metabolism, citrate cycle (TCA cycle), pyruvate metabolism, pentose phosphate pathway, fatty acid metabolism, fatty acid degradation, fatty acid biosynthesis, lipid metabolism, biosynthesis of antibiotics, biosynthesis amino acids, terpenoid backbone biosynthesis, synthesis and degradation of ketone bodies, DNA replication, biotin metabolism, transporter, ABC transporter.

The reconstruction of the involvement of each transcript through KEGG identifier mapping exhibited 216 DEGs (40% of total DEGs) mapped into 6 branches with 594 pathways (Figure 3). In total, among the KEGG-annotated sequences, 164 were grouped under “metabolism” category (76% of total DEGs assigned to KEGG identifiers), 34 under “environmental information processing” (16%), 22 under “genetic information processing” (10%), 16 under “cellular processes” identifier (7%), and 4 under “organismal systems” (2%). More in detail, 70% were classified into “metabolic pathway,” 29% into “microbial metabolism in diverse environments,” 24% into “fatty acid metabolism/biosynthesis/degradation,” 22% into “biosynthesis of secondary metabolites,” 16% into “carbon metabolism,” and 9% into “transporter” (Figure 3).

Overall, the transcriptome showed DEGs associated with stress response, osmotic regulation, central metabolism, and oxidative metabolism of aromatic hydrocarbons. On the other hand, the analyses also showed that 26% of the positively activated genes and 32% of the down-regulated genes were preliminary annotated as hypothetical proteins with unknown functions (Supplementary Table S1).

R7 transcriptome analysis evidenced 14 up-regulated transcripts possibly related to stress response, including a possible universal stress protein and a ClpB protein. In particular, a region containing genes encoding for chaperone proteins (DnaK and CbpA), heat shock proteins (GrpE) and thioredoxin gene cluster were up-regulated with a range of 2.3 – 3.7 fold-change (Supplementary Tables S1, S2 – Stress response). On the other hand, 18 transcripts were down-regulated, including 3 copper chaperon encoding genes, a multicopper oxidase, a copper translocating ATPase, 2 universal stress proteins, one heat shock protein (family chaperone, GroEL), one catalase, and a hypothetical protein associated to bacteriocin-protection were down-regulated with a fold-change varying from -2.3 to -6.5 (Supplementary Tables S1, S2 – Stress response).

Moreover, results revealed up-regulated genes related to osmotic stress regulation such as one choline dehydrogenase (EC 1.1.99.1) (BetA) among the 9 present in R7 genome and one L-proline/glycine betaine transporter (TC 2.A.1.6.4) (ProP) that were predicted to be involved, respectively, in the choline oxidation and proline transport. As previously hypothesized for R7 strain (Cappelletti et al., 2016), R7 also possesses glycine betaine/carnitine/choline ABC transporter/permease protein ProU complex (ProV, ProW, and ProX), an aldehyde dehydrogenase (associate with a “glycine betaine biosynthetic process from choline” GO), and glycine betaine ABC transport permease protein (TC 3.A.1.12.1) that were down-regulated (Supplementary Tables S1, S2 – Osmotic stress).

Overall, 65 different transport proteins appeared differentially regulated, among which 61% were down-regulated. According to the annotation, these transport systems are related to (i) alternative carbon source transporters, (ii) balance of osmotic stress and (iii) metal ions transporters [i.e., lead, cadmium, zinc, and mercury transporting ATPase (EC 3.6.3.3) (EC 3.6.3.5) or copper-translocating P-type ATPase (EC 3.6.3.4) enzymes]. Among the activated transport systems, the biotin integral membrane systems and the ferrous iron transport permeases (e*feU* and *efeO*) showed a slight activation of around 3.6 and 2.3–2.5 fold-change, respectively (Supplementary Tables S1, S2 – Transporters).

The transcriptome also showed the activation of 10 chromosomal CDSs with an average expression value of 3.3-fold and one of around -4.1-fold that are attributable to inositol catabolism due to GO association with “inositol catabolic process” (Supplementary Tables S1, S2 – Inositol catabolism).

Stress endurance related to the presence of organic xenobiotics generates cell instability consisting of a slowdown of the synthesis of tricarboxylic acid cycle enzymes (Domínguez-Cuevas et al., 2006). Actually, after R7 exposure to *o*-xylene, several tricarboxylic acid enzymes were repressed, such as three glutamate permeases, one dicarboxylate transporter for fumarate, L-malate, D-malate, succinate, one putative sodium:dicarboxylate symporter TctB, and TctC citrate transporters, one citrate lyase *beta* chain (EC 4.1.3.6) (*aceA*), a probable pyruvate carboxylase (*ppc*), and a 6-phosphogluconate dehydrogenase decarboxylating (EC 1.1.1.44) (Supplementary Tables S1, S2 – TCA cycle). However, comparing R7 gene expression to *Pseudomonas putida* KT2440 Krebs cycle genes (Domínguez-Cuevas et al., 2006), results showed that gene encoding for a glucose-6-phosphate dehydrogenase (*zwf-1*), two pyruvate dehydrogenases E1 component (*aceE*), two malate dehydrogenases (*mdh1* and *mdh2*), a isopropylmalate synthase (*glcB*), and a succinate-semialdehyde dehydrogenase [NADP+] (EC 1.2.1.16) (*gabD*) exhibited expression values ranging from 3.2 to 4.8 fold-change (Supplementary Tables S1, S2 – TCA cycle).

### Analysis of Specific Genetic Determinants of *R*. *opacus* R7 Induced by *o*-Xylene by RNA-Seq

*o*-Xylene induced the expression of several *R*. *opacus* R7 genes putatively involved in aromatic hydrocarbon degradation according to the GO and the KEGG analysis. Indeed, a high number of DEGs were assigned to the GO category namely “aromatic compound catabolic process” and specifically to “oxygenase,” “monooxygenase,” and “dioxygenase” (Figure 1). Among the others, the genes encoding for the following enzymatic classes showed high transcriptional levels: dioxygenases, monooxygenases, hydroxylase, dehydrogenases, reductases, and aldolases (Supplementary Table S1). Considering the DEGs mapped in KEGG database, few DEGs are also assigned to the “xylene degradation” and “toluene degradation” (Figure 3). All the above mentioned genes with a putative role in the aromatic hydrocarbon degradation were considered and analyzed in order to identify the genetic determinants involved in the *o*-xylene metabolic pathway.

Among 129 oxygenases/hydroxylases within the R7 genome that are putatively involved in organic compound degradation (Di Gennaro et al., 2014), 17 were up-regulated when R7 cells grew in the presence of *o*-xylene (Supplementary Table S3). The highly up-regulated dioxygenases (7.8–4 fold-change values), such as AII11493 (*akbA1a*) and AII10987 (*padAa*) were analyzed by manual sequence alignments and the analysis of conserved amino acids residues. These sequences were compared against another rhodococcal dioxygenase that is known to have a role in the aromatic hydrocarbon degradation and specifically in the BTEX compounds. *Rhodococcus*. sp. DK17 AkbA1a protein sequence (Kim et al., 2004) was used as a reference and the comparison showed that six on seven characteristic domains of DK17 AkbA1a were conserved among the three sequences (i) “Rieske RO *alpha* NDO,” Rieske non-heme iron oxygenase (RO) family; (ii) “RHO *alpha* C NDO-like,” C-terminal catalytic domain of the oxygenase *alpha* subunit of naphthalene 1,2 dioxygenase; (iii) “benzo 1,2 benA,” benzoate 1,2 dioxygenase, large subunit; (iv) “HcaE,” phenylpropionate dioxygenase related to ring-hydroxylating dioxygenase; (v) “Rieske,” Rieske [2Fe-2S] domain; and (vi) “Ring hydroxyl A,” ring hydroxylating *alpha* subunit) and only PadAa was characterized by an eighth specific domain (“anthran 1,2 A,” anthranilate 1,2 dioxygenase, large subunit).

Moreover, the GO mapping assigned the “xylene catabolic process” category only to *akbA1a* gene, whilst the “biphenyl 2,3-dioxygenase activity” category to *padA* gene (Figure 1).

Considering the AII11493 dioxygenase system, six genes located on the pPDG5 plasmid encoding for the Akb dioxygenase system appeared highly up-regulated (7.4 fold-change). Specifically, *akbA1a* and *akbA2a* genes encoding, respectively, for the large and the small subunit of the ethylbenzene dioxygenase (AkbA1a and AkbA2a), *akbA3* gene for the ferredoxin component (AkbA3), *HP* sequence for a hypothetical protein (HP) of unknown function, *akbA4* gene for the ferrodoxin reductase component (AkbA4), and *akbB* gene coding for the dihydrodiol dehydrogenase (AkbB). Moreover, an expression of 7.4-fold was observed for *akbCDEF* genes coding for the complete *meta*-cleavage enzymes of the lower pathway. They are allocated on the pPDG2 plasmid, including a *meta*-cleavage dioxygenase (AkbC), a *meta*-cleavage hydrolase (AkbD), a hydratase component (AkbE), and an aldolase (AkbF). The high expression of these gene clusters supported our previous results regarding the activation of the *akb* gene clusters for the complete metabolism of *o*-xylene (Orro et al., 2015; Di Canito et al., 2018; Supplementary Table S3).

Although R7 *akb* genes are the undisputed oxidative system for *o*-xylene, other dioxygenases were revealed to be differentially expressed. Among the highly DEGs, genes allocated on the pPDG2 plasmid encoding for a putative phthalate dioxygenase system (*pad* genes) showed an expression of 7.8 fold-change (Supplementary Table S3). The deduced amino acid sequence of each CDS was compared to *R*. *jostii* RHA1 phthalate dioxygenase (Patrauchan et al., 2005; Hara et al., 2007). The alignments showed an amino acid identity, respectively, of 84% and 87% for the *alpha* (*padAa*) and the *beta* (*padAb*) subunits of the phthalate 3,4-dioxygenase (Rieske 2Fe-2S domain-containing protein); of 76% and 61% for the hypothetical protein (*padAc*) and the ferredoxin reductase (*padAd*), respectively; for the phthalate dihydrodiol dehydrogenase (*padB*) and the 3,4-dihydroxyphthalate decarboxylase (annotated as uncharacterized protein) (*padC*) showed an amino acid identity of 85% and 83%, respectively (Supplementary Table S3).

Moreover, in order to assess the differences between the two mainly expressed dioxygenases, the sequences of the catalytic subunits PadA and AkbA were aligned and they shared 34% amino acid identity (50% nucleotide identity), despite the conserved domains.

The genome analysis of *pad* gene region also showed a transcriptional regulator of IclR family located upstream of the *padAa* gene. However, the transcriptome analysis did not exhibit this regulatory gene among the differentially regulated sequences (Supplementary Table S1). On the contrary, the *akbT* gene encoding for a response regulator two-component system located upstream of the *akbA1a* gene was induced by *o*-xylene with an expression value of 3.78 fold-change (Supplementary Tables S1, S3).

The analysis of other R7 oxygenases showed that also the dioxygenases putatively involved in the central (or peripheral) aromatic metabolism were up-regulated during *o*-xylene degradation. We detected *catA*, encoding for a catechol 1,2-dioxygenase (EC:1.13.11.1) among the six in R7 genome, *pcaH* and *pcaG* encoding for *alpha* and *beta* subunits of the protocatechuate 3,4-dioxygenase (EC 1.13.11.3), respectively; *alpha* and *beta* subunits of the benzoate dioxygenase (EC:1.14.12.10; 1.14.12.-) (Supplementary Tables S1, S3). Concerning monooxygenases, three cyclohexanone monooxygenases (CzcO1, CzcO6, and CzcO7) (EC 1.14.13.22), an alkanal monooxygenase *alpha*-chain (EC 1.14.14.3) (LuxA2), a predicted monooxygenase (RutA), and two flavin reductase (NADH) monooxygenases (FmoB) (a hypothetical protein belonging to a nitriloacetate monooxygenase family and a nitriloacetate monooxygenase component B) were up-regulated with a fold-change of 3.5, 4.9, 6.6, 3.3, 2.7, 9.3, and 2.4, respectively (Supplementary Tables S1, S3). The latter is located downstream a gene encoding for a 2,3-dihydroxybiphenyl 1,2-dioxygenase (EC 1.13.11.39) and they could be involved in the lower metabolism of aromatic compounds. Conversely, seven different monooxygenases appeared down-regulated with a fold-change varying from -2.3 to -6.6; for instance, the genes belonging to *prm* gene cluster that was previously thoroughly analyzed (Di Canito et al., 2018; Supplementary Tables S1, S3).

In summary, *R*. *opacus* R7 exhibited a great array of DEGs converging toward the *o*-xylene metabolism.

### RT-qPCR Analysis of Selected *R*. *opacus* R7 Genes From RNA-Seq

**FIGURE 4:**
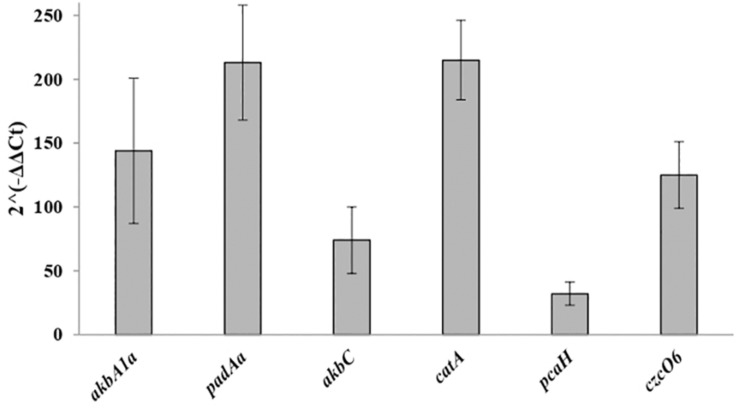
Expression levels of R7 selected DEGs by RT-qPCR analysis. The selected genes are *akbA1a*, *padAa*, *akbC*, *catA*, *pcaH*, and *czcO6* genes encoding for the ethylbenzene dioxygenase, the phthalate 3,4-dioxygenase, the *meta*-cleavage dioxygenase, the catechol 1,2-dioxygenase, the protocatechuate 3,4-dioxygenase, and the cyclohexanone monooxygenase.

In order to confirm the transcriptome accuracy and to support our findings on the main dioxygenase systems that could be potentially involved in *o*-xylene degradation, RT-qPCR experiments were performed. The transcription levels of the catalytic subunits of the candidate oxygenases such as the ethylbenzene dioxygenase, the phthalate 3,4-dioxygenase, the *meta*-cleavage dioxygenase, the catechol 1,2-dioxygenase, the protocatechuate 3,4-dioxygenase, and the cyclohexanone monooxygenase encoded, respectively, by *akbA1a*, *padAa*, *akbC*, *catA*, *pcaH*, and *czcO6* genes were measured after R7 cell growth in the presence of *o*-xylene or malate as shown in Figure 4. The gene expression fold-change between RNA-seq and RT-qPCR demonstrated a substantial correlation with virtually identical trends for each selected gene. Therefore, our transcriptomic findings were confirmed by the trends reported by RT-qPCR analyses, suggesting the reliability and accuracy of the RNA-seq expression analysis.

## Discussion

The present study reports for the first time the transcriptome analysis of a member of *Rhodococcu*s genus grown in the presence of *o*-xylene which is the most recalcitrant compound among xylenes. We have developed an RNA-seq approach to unveil *R*. *opacus* strain R7 genetic determinants encoding for candidate enzymes involved in the *o*-xylene degradation.

Few members of *Rhodococcus* genus are able to grow on *o*-xylene as the sole source of carbon and energy. Moreover, the genetic mechanisms of *o*-xylene oxidation are not clear. Two main routes are described in rhodococcal strains: (i) the oxidation of the methyl group of *o*-xylene to form 2-methylbenzylalcohol which is subsequently metabolized to form 3-methylcatechol as reported in *Rhodococcus* sp. strain B3 and *R. opacus* TKN14 (Bickerdike et al., 1997; Maruyama et al., 2005); (ii) the ring-oxidation leading to the 3,4-DMC as reported in *Rhodococcus* sp. strain DK17 (Kim et al., 2004).

Previous work demonstrated that *R. opacus* R7 can degrade the *ortho*-isomer producing the 2,3- and 3,4-DMPs as the main intermediates (Di Gennaro et al., 2001). R7 was supposed to be able to perform both dioxygenation and monooxygenation reactions to completely mineralize the *ortho*-isomer. In other respects, Kim and co-authors showed the formation of phenolic intermediates deriving from the dehydration to dihydrodiol through the action of the Akb oxygenase system (Kim et al., 2004).

**FIGURE 5:**
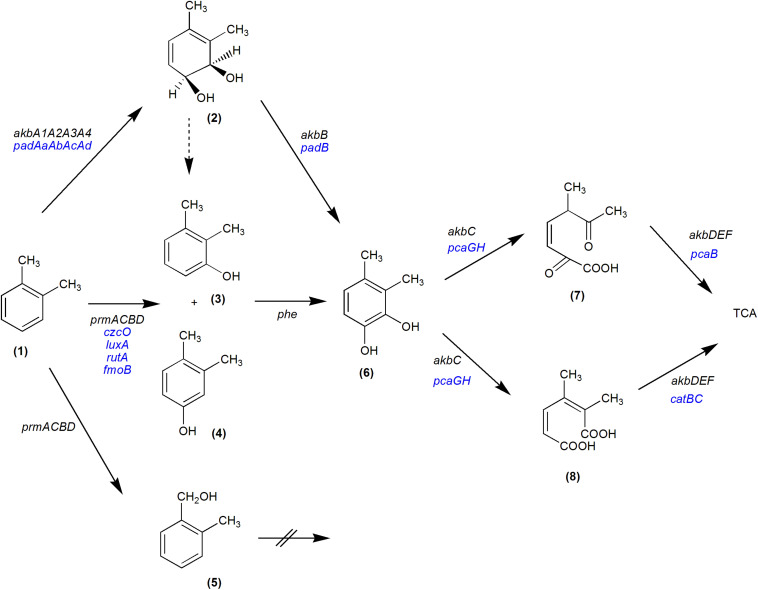
Proposed metabolic pathways of *R*. *opacus* R7 for *o*-xylene degradation. The black genes represent the confirmed genes involved in the *o*-xylene pathway: *akbA1A2A3A4*, multicomponent *o*-xylene dioxygenase; *akbB*, dihydrodiol dehydrogenase; *akbC*, *meta*-cleavage dioxygenase; *akbD*, a *meta*-cleavage hydrolase; *akbE*, a hydratase component; *akbF*, aldolase; *prmACBD*, monooxygenase; *phe*, phenol hydroxylase. The blue genes were identified on the basis of transcriptomic data and are potentially involved in the *o*-xylene pathway: *padAaAbAcAd*, multicomponent phthalate dioxygenase; *padB*, phthalate dihydrodiol dehydrogenase; *czcO*, cyclohexanone monooxygenases; *luxA*, alkanal monooxygenase *alpha*-chain; *rutA*, predicted monooxygenase; *fmoB*, flavin reductase (NADH) monooxygenases; *catA*, cathecol 1,2-dioxygenase; *catB*, muconate cycloisomerase; *pcaB*, 3-carboxy-cis,cis-muconate cycloisomerase; *pcaG* and *pcaH*, *alpha* and *beta* subunits of the protocatechuate 3,4-dioxygenase, respectively. Dashed arrow indicates a spontaneous dehydration. (1) *o*-xylene; (2) *cis*-3,4-dihydrodiol; (3) 2,3-dimethylphenol; (4) 3,4-dimethylphenol; (5) 2-methylbenzylalcohol; (6) 3,4-dimethylcatechol; (7) 5-methyl-2,6-dioxohept-3-enoic acid; (8) 2,3-dimethylhexa-2,4-dienedioic acid.

The analysis of R7 transcriptome during growth on *o*-xylene compared to malate condition showed that the principal genes activated are those of the *akb* system. The amino acid sequence of each CDS belonging to *akb* gene cluster appeared homologous to the correspondent of Akb oxygenase system of *Rhodococcus* sp. strain DK17 (Kim et al., 2004). Therefore, the transcriptomic data demonstrated that R7 oxidation mechanism of *o*-xylene is through the action of the Akb ring-hydroxylating dioxygenase system. These results are in accordance with previous data showing that *akb* genes are the main genetic determinants for dioxygenation route in R7 *o*-xylene degradation (Di Canito et al., 2018). Moreover, among up-regulated *akb* genes, the transcriptome exhibited *akbT* and HP genes encoding for the transcriptional regulator belonging to NarL/FixJ family containing REC and HTH domains and the hypothetical protein that according to manual blast and GO analyses is a sensor kinase. This result indicates that *o*-xylene is able to activate and to up-regulate the regulative system of *akb* genes (Figure 5).

Surprisingly, the transcriptome analysis of R7 grown in the presence of *o*-xylene showed another activated dioxygenase system, the *pad* system. Among the up-regulated *pad* genes, no genes encoding for a regulatory protein was significantly expressed. Thus, we hypothesized that the specific transcriptional regulator is always activated under both malate and *o-*xylene conditions, thus the *pad* system is generally activated, and *pad* genes recognize the *o*-xylene as an inducer. To better clarify, the deduced amino acid sequence of *pad* dioxygenase was compared to the most known *Rhodococcus* dioxygenases involved in aromatic hydrocarbon degradation. The comparison highlighted that the diverse rhodococcal dioxygenase systems present conserved and shared domains (EC 1.14.12-). However, the alignments showed that the Pad dioxygenase was homologous around 84% respect to *R*. *jostii* RHA1 phthalate dioxygenase (Patrauchan et al., 2005; Hara et al., 2007).

Among the numerous extradiol dioxygenases that can be activated during the oxidation of *o*-xylene, the catechol 1,2-dioxygenase (*cat* genes) and protocatechuate 3,4-dioxygenase (*pca* genes) were expressed and involved in the central metabolism. Accordingly, Gonçalves and coworkers (Gonçalves et al., 2006) reported that multiple numbers of extradiol dioxygenases can be present and activated during the same oxidation metabolism. Respect to this, we could completely reconstruct the *o*-xylene degradation pathway in R7 (Figure 5).

However, the RNA-seq analysis of *R*. *opacus* R7 exhibited other monooxygenases/hydroxylases with a significant value of fold-change during growth on *o*-xylene, such as the cyclohexanone monooxygenases. Among the others, we observed the down-regulation of the Prm monooxygenase (*prmACBD*), which was identified and characterized in our previous work for the ability to slowly transform the *o*-xylene in intermediates such as 3,4-dimethylphenol and 2-methylbenzylalcohol by cloning and heterologous expression experiments (Di Canito et al., 2018). This could appear in contrast, nonetheless, the Prm monooxygenase system was switched off only in the condition tested i.e., the exponential growth, but it is indubitable its ability in performing ring- and methyl-oxidations. A possible explanation of these results is that *prm* genes can induce an increase of phenol levels, but under the condition tested, the expression levels were not sufficient to promote such activation in favor of other leading routes such as the *o*-xylene dioxygenase route. Indeed, the change of the gene expression levels is highlighted in several bacteria reported in literature (Arenghi et al., 1999; Janaszak et al., 2009; Yoneda et al., 2016).

Finally, RNA-seq data could suggest that Akb system is mainly involved and regulated and confirmed that R7 possesses a redundancy of other sequences encoding for oxygenases/hydroxylases converging in *o*-xylene oxidation (Figure 5).

Likewise, the presence of organic xenobiotics can result in a slowdown of the synthesis of tricarboxylic acid cycle enzymes that is reversed by the utilization of the toxic compound as a carbon source (Domínguez-Cuevas et al., 2006). This was confirmed by R7 RNA-seq data that showed diverse enzymes that are likely implicated in this role and each one appeared inhibited.

Simultaneously, it has been reported that *o*-xylene can cause oxidative stress in bacteria (Domínguez-Cuevas et al., 2006). In *Pseudomonas* strains, the mechanism to respond to such stress can be observed at the level of cell envelope, lipid production and stress response (Pinkart et al., 1996; Baumgarten et al., 2012). Also, Gram-positive bacteria activate mechanisms for organic solvent tolerance such as the induction of general stress regulon or the production of organic solvent emulsifying or deactivating enzymes (Torres et al., 2011). The RNA-seq approach proved that the *o*-xylene entrance in R7 cells generated physiological changes and adaptation. Indeed, R7 transcriptome showed that several DEGs are related to stress response, many of which were previously predicted to have this function (Cappelletti et al., 2016).

Interestingly, *o*-xylene metabolism entailed alteration of R7 cell osmolarity as *Pseudomonas putida* KT2440 (Domínguez-Cuevas et al., 2006). Several R7 genes associated to osmotic stress were observed differentially regulated (both negatively and positively expressed) and connected to L-proline/glycine betaine transport. The diverse response of these transport systems to osmotic stress could be explained by the two-step adaptation mechanism observed in *Bacillus subtilis* (Kappes et al., 1996). This mechanism requires the accumulation of proline under high-osmolarity growth conditions through *de novo* synthesis that is a slow process. Likewise, the faster accumulation of glycine betaine as osmoprotectant is an efficient response to osmolarity and it can be synthesized from choline in case of unobtainability (Kappes et al., 1996). Therefore, different transport systems related to osmotic stress were inevitably activated and regulated.

Generally, the cell instability can likely be provoked by the presence of organic xenobiotics generally induced by organic solvents and eventually result in a slowdown of the synthesis of tricarboxylic acid cycle enzymes. This stress condition can only be compensated when the toxic compound is used as a carbon source (Domínguez-Cuevas et al., 2006). Although *o*-xylene metabolism generated a general stress observed in R7 cells, this strain maintained the principal productive step of aerobic metabolism related to Krebs cycle. Indeed, Krebs cycle enzymes were activated in R7 strain. This is in contrast to *Pseudomonas putida* KT2440 transcriptome obtained after exposition to metabolizable toluene and non-metabolizable *o*-xylene. KT2440 reported a slowdown of Krebs cycle enzymes in presence of both compounds and not in presence of 3-methylbenzoate used as control (Domínguez-Cuevas et al., 2006).

Besides, genes encoding enzymes for inositol catabolism were up-regulated. Inositol is a component of mycothiol involved in membrane and cell wall processes in most actinobacteria. Notably, the inositol catabolism is not only correlated to the membrane and the cell wall constituents, but inositol could be used as a cellular redox regulator (Michell, 2011).

In conclusion, coupling genomic data and an *omic*-approach viz the RNA-seq allowed unveiling the multiplicity of *R. opacus* R7 genetic determinants for *o*-xylene degradation. This high-throughput technique contributed mostly to depict the degradative pathway redundancy that ensures *Rhodococcus* members functional versatility, overall robustness, and genome plasticity (Alvarez, 2019). It also provided hints about the sophisticated regulatory network behind *Rhodococcus* catabolic pathways that still need to be explored.

More knowledge has been also acquired on the complexity of background machinery related to the stress response that is not always easily assessable due to the fast reassignment of transcriptional elements of dispensable functions and required for stress tolerance in the presence of environmental widespread contaminants (Domínguez-Cuevas et al., 2006).

## Data Availability Statement

The datasets generated for this study can be found in the European Nucleotide Archive with the following accession number: PRJEB38098.

## Author Contributions

AD carried out the experiments that were planned and conceived with JZ and PD. JZ contributed to the analysis of the data and wrote the manuscript. AM and AO analyzed the bioinformatic data. PD and LM financially supported the project and provided the critical feedback. PD helped in shaping the manuscript. All authors provided critical feedback and contributed to the final manuscript.

## Conflict of Interest

The authors declare that the research was conducted in the absence of any commercial or financial relationships that could be construed as a potential conflict of interest.
