# Supplementary material for: Transcriptomic Analysis of Rhodococcus opacus R7 Grown on o-Xylene by RNA-Seq
Source: Front Microbiol. 2020 Aug 12;11:1808. doi: 10.3389/fmicb.2020.01808 (PMC7434839; doi:10.3389/fmicb.2020.01808)
Supplement: Supplementary file 2 [file Table_2.docx]

**Supplementary Table S2** List of *R. opacus* R7 DEGs after RNA-seq analysis. Genes related to stress response, osmotic stress, inositol catabolism, transporters, and TCA cycle are listed together with their relative expression values.

| **ID NCBI** | **Gene Name** | **Location** | **Function** | **log2(fold_change)** | **GO** | **KEGG** | **Uniprot ID** | **Score** | **e-value** |
| --- | --- | --- | --- | --- | --- | --- | --- | --- | --- |
| Stress response | | | | | | | | | |
| AII07200 | *uspA1*  peg.3705 | Chromosome | possible universal stress protein | 3.7 | - | - | A0QZZ6 | 146 | 8.86E-12 |
| AII03329 | *clpB*  peg.7295 | Chromosome | ClpB protein | 3.2 | Cytoplasm, ATP binding, protein metabolic process, protein refolding, response to heat | - | Q826F2 | 3400 | 0 |
| AII03333 | *dnaK* peg.7299 | Chromosome | Chaperone protein DnaK | 3.2 | ATP binding, protein folding, unfolded protein binding | RNA degradation | Q826F6 | 2521 | 0 |
| AII03331 | *cbpA*  peg.7297 | Chromosome | DnaJ-class molecular chaperone CbpA | 3.2 | Cytoplasm, DNA binding, unfolded protein binding, protein refolding, nucleoid, bent DNA binding, chaperone cofactor-dependent protein refolding | - | Q83CJ2 | 536 | 2.36E-65 |
| AII03332 | *GrpE* peg.7298 | Chromosome | Heat shock protein GrpE | 3.2 | Cytoplasm, protein folding, chaperone binding, protein homodimerization activity, adenyl-nucleotide exchange factor activity | - | Q826F5 | 488 | 1.09E-61 |
| AII03328 | *trxA* - peg.7294 | Chromosome | thioredoxin | 3.2 | Cytoplasm, cytosol, cell, cell redox homeostasis, protein disulfide oxidoreductase activity, protein-disulfide reductase activity, oxidoreductase activity, acting on a sulfur group of donors, disulfide as acceptor, glycerol ether metabolic process | - | P43785 | 270 | 2.66E-30 |
| AII03327 | *trxB* -peg.7292 | Chromosome | Thioredoxin reductase (EC 1.8.1.9) | 3.2 | Cytoplasm, thioredoxin-disulfide reductase activity, removal of superoxide radicals | - | P23160 | 349 | 1.54E-35 |
| AII11304 | *copA -*peg.9315 | Chromosome | Copper chaperone | -3.8 | Cytoplasm, copper ion binding, metal ion transport | - | Q6GDP0 | 121 | 4.75E-09 |
| AII11312 | *copA -*peg.9331 | pPDG3 | Copper chaperone | -4.0 | Cytoplasm, copper ion binding, metal ion transport | - | Q79ZY4 | 101 | 3.57E-06 |
| AII11320 | *copA -*peg.9341 | pPDG3 | Copper chaperone | -5.9 | Cytoplasm, copper ion binding, metal ion transport | - | Q79ZY4 | 119 | 4.42E-09 |
| AII11185 | *mmcO* - peg.9170 | pPDG3 | Multicopper oxidase | -4.2 | Plasma membrane, copper ion binding, periplasmic space, ferroxidase activity | Porphyrin and chlorophyll metabolism | I6WZK7 | 1074 | 9.4202E-141 |
| WP_128644070 | *copB*  peg.9326 | pPDG3 | Hypothetical protein- copper-translocating P-type ATPase | -4.1 | ATP binding, plasma membrane, integral component of membrane, cytosol, extracellular matrix organization, nucleus, chaperone binding, perinuclear region of cytoplasm, cell, endoplasmic reticulum , Golgi apparatus, perikaryon, membrane, neuron projection, trans-Golgi network, microvillus, removal of superoxide radicals, neuronal cell body, ion transmembrane transport, membrane raft, late endosome, apical plasma membrane, copper ion binding, collagen fibril organization, phagocytic vesicle membrane, cellular response to hypoxia, cellular response to iron ion, response to zinc ion, tryptophan metabolic process, pigmentation, dopamine metabolic process, negative regulation of catalytic activity, positive regulation of catalytic activity, cell leading edge, liver development, locomotory behavior, copper ion transport, mitochondrion organization, regulation of gene expression, copper ion transmembrane transporter activity, copper ion import, secretory granule, negative regulation of iron ion transmembrane transport, copper transmembrane transporter activity, phosphorylative mechanism, lactation, serotonin metabolic process, cellular response to amino acid stimulus, cellular copper ion homeostasis, copper-dependent protein binding, cuprous ion binding, superoxide dismutase copper chaperone activity, catecholamine metabolic process, cellular response to antibiotic , cellular response to cadmium ion, cellular response to cobalt ion, cellular response to copper ion, cellular response to lead ion, copper ion export, detoxification of copper ion, elastin biosynthetic process, epinephrine metabolic process, norepinephrine metabolic process, peptidyl-lysine modification, positive regulation of cell size, positive regulation of oxidoreductase activity, regulation of cytochrome-c oxidase activity, regulation of oxidative phosphorylation, response to iron(III) ion, response to manganese ion | Platinum drug resistance, Mineral absorption | Q04656 | 72 | 0.438822 |
| AII08924 | *uspA1 -* peg.5659 | Chromosome | Universal stress protein family | -3.5 | ATP binding | - | P9WFD1 | 313 | 1.21E-32 |
| AII08926 | *uspA1 -* peg.5661 | Chromosome | Universal stress protein family | -3.5 | ATP binding | - | P64922 | 441 | 1.49E-51 |
| AII03491 | *groEL* - peg.7519 | Chromosome | Heat shock protein 60 family chaperone GroEL | -4.3 | ATP binding, protein refolding | RNA degradation | Q0SJK2 | 2538 | 0 |
| AII03501 | *katE*  peg.7530 | Chromosome | Catalase (EC 1.11.1.6) | -6.5 | Metal ion binding, catalase activity, heme binding, hydrogen peroxide catabolic process, response to oxidative stress | Tryptophan metabolism, Glyoxylate and dicarboxylate metabolism, Metabolic pathways, Biosynthesis of secondary metabolites, Biosynthesis of antibiotics, Carbon metabolism | Q9ZN99 | 1890 | 0 |
| AII04382 | *ydeI* or *ompD* - peg.677 | Chromosome | FIG00996025: hypothetical protein - Bacteriocin-protection, YdeI or OmpD-Associated | -2.6 | Metal ion binding, response to oxidative stress, cytosol, peptide-methionine (R)-S-oxide reductase activity, protein repair | - | Q9M0Z5 | 77 | 0.29601 |
| Osmotic stress | | | | | | | | | |
| AII08178 | *betA*  peg.4849 | Chromosome | Choline dehydrogenase (EC 1.1.99.1) | 2.6 | Flavin adenine dinucleotide binding, oxidoreductase activity, acting on CH-OH group of donors | - | Q47944 | 788 | 1.22E-97 |
| AII09834 | *proP*  peg.6630 | Chromosome | L-Proline/Glycine betaine transporter ProP | 2.3 | Integral component of plasma membrane, symporter activity | - | P40862 | 900 | 5.5E-115 |
| AII08571 | *proX*  peg.5300 | Chromosome | L-proline glycine betaine binding ABC transporter protein ProX (TC 3.A.1.12.1) | -4.4 | Amino acid transport, transmembrane transporter activity, ATP-binding cassette (ABC) transporter complex | ABC transporters | Q45462 | 218 | 6.6E-19 |
| AII08572 | *proW*  peg.5301 | Chromosome | Glycine betaine ABC transport system permease protein | -3.4 | Plasma membrane, integral component of membrane, transmembrane transport | - | Q9KHT6 | 161 | 5.82E-12 |
| AII08573 | *proW*  peg.5302 | Chromosome | L-proline glycine betaine ABC transport system permease protein ProW (TC 3.A.1.12.1) | -3.4 | Plasma membrane, integral component of membrane, transmembrane transport | - | Q9KHT8 | 153 | 4.1E-11 |
| AII08574 | *proV* peg.5303 | Chromosome | L-proline glycine betaine ABC transport system permease protein ProV (TC 3.A.1.12.1) | -3.4 | ATP binding, ATPase activity, amino acid transport, membrane, glycine betaine transport | ABC transporters | O34992 | 739 | 3.91E-94 |
| AII08040 | *betA2*  peg.4677 | Chromosome | Aldehyde dehydrogenase (EC 1.2.1.3) - choline | -5.4 | Metal ion binding, betaine-aldehyde dehydrogenase activity, glycine betaine biosynthetic process from choline | Metabolic pathways, Glycine, serine and threonine metabolism | P71016 | 584 | 3.40099E-68 |
| Transporters | | | | | | | | | |
| AII11307 | *copA* peg.9319 | pPDG3 | Lead, cadmium, zinc and mercury transporting ATPase (EC 3.6.3.3) (EC 3.6.3.5); Copper-translocating P-type ATPase (EC 3.6.3.4) | -3.9 | ATP binding, plasma membrane, integral component of membrane, copper ion binding, copper ion transport, ATPase-coupled cation transmembrane transporter activity | - | Q4A0G1 | 1401 | 0 |
| AII11318 | *copB* peg.9339 | pPDG3 | Lead, cadmium, zinc and mercury transporting ATPase (EC 3.6.3.3) (EC 3.6.3.5); Copper-translocating P-type ATPase (EC 3.6.3.4) | -5.9 | [GO:0046872] metal ion binding [GO:0005524] ATP binding [GO:0005886] plasma membrane [GO:0016021] integral component of membrane [GO:0030001] metal ion transport [GO:0019829] ATPase-coupled cation transmembrane transporter activity | - | P46840 | 1203 | 2.172E-153 |
| AII04546 | *actVA1*  peg.8446 | pPDG1 | Integral membrane transport protein | 3.6 | Plasma membrane, integral component of membrane, transmembrane transport | - | P9WJW6 | 271 | 5.32E-28 |
| WP_128638966 | *actVA1*  peg.8447 | pPDG1 | Integral membrane transport protein | 3.6 | Extracellular region, plasma membrane, integral component of membrane, transmembrane transport | - | P9WJW7 | 153 | 5.91E-12 |
| AII05276 | *efeU* peg.1625 | Chromosome | Ferrous iron transport permease EfeU | 2.3 | Iron ion homeostasis, high-affinity iron permease complex, iron ion transmembrane transporter activity | - | Q8FJ36 | 304 | 2E-31 |
| AII05277 | *efeO* eg.1626 | Chromosome | Ferrous iron transport periplasmic protein EfeO, contains peptidase-M75 domain and (frequently) cupredoxin-like domain | 2.5 | Periplasmic space | - | Q8XAS6 | 555 | 1.53E-66 |
| Inositol catabolism | | | | | | | | | |
| 1I - AII07201 | *iolG* peg.3706 | Chromosome | Myo-inositol 2-dehydrogenase (EC 1.1.1.18) | 3.2 | Oxidoreductase activity | - | P55480 | 518 | 1.93E-60 |
| AII07202 | *iolG*  peg.3707 | Chromosome | Myo-inositol 2-dehydrogenase (EC 1.1.1.18) | 3.8 | Oxidoreductase activity, NADPH regeneration, inositol 2-dehydrogenase activity, inositol metabolic process | Metabolic pathways, Biosynthesis of secondary metabolites, Biosynthesis of antibiotics, Microbial metabolism in diverse environments, Streptomycin biosynthesis, Inositol phosphate metabolism | Q9WYP5 | 515 | 1.07E-61 |
| AII09778 | *iolA*  peg.6569 | Chromosome | Methylmalonate-semialdehyde dehydrogenase [inositol] (EC 1.2.1.27) | 3.3 | Malonate-semialdehyde dehydrogenase (acetylating) activity, methylmalonate-semialdehyde dehydrogenase (acylating) activity, beta-alanine biosynthetic process, thymine catabolic process, valine catabolic process, valine metabolic process | Valine, leucine and isoleucine degradation, beta-Alanine metabolism, Inositol phosphate metabolism, Propanoate metabolism, Metabolic pathways, Carbon metabolism, | Q9I702 | 1340 | 0 |
| AII09779 | *tpiA*  peg.6570 | Chromosome | Glyceraldehyde-3-phosphate ketol-isomerase (EC 5.3.1.1) | 3.3 | - | - | P42418 | 596 | 5.15E-75 |
| AII09780 | *iolG* peg.6571 | Chromosome | Myo-inositol 2-dehydrogenase (EC 1.1.1.18) | 3.3 | Inositol catabolic process, inositol 2-dehydrogenase activity | Metabolic pathways, Microbial metabolism in diverse environments, Biosynthesis of secondary metabolites, Inositol phosphate metabolism, Biosynthesis of antibiotics, Streptomycin biosynthesis | Q0SH07 | 1513 | 0 |
| AII09781 | *iolE*  peg.6572 | Chromosome | Inosose dehydratase (EC 4.2.1.44) | 3.3 | Manganese ion binding, inositol catabolic process, myo-inosose-2 dehydratase activity | Inositol phosphate metabolism, Metabolic pathways, Microbial metabolism in diverse | Q4A8D5 | 223 | 9.31E-20 |
| AII09782 | *iolD*  peg.6573 | Chromosome | Epi-inositol hydrolase (EC 3.7.1.-) | 3.3 | Magnesium ion binding, thiamine pyrophosphate binding, inositol catabolic process, 3D-(3,5/4)-trihydroxycyclohexane-1,2-dione hydrolase activity | Inositol phosphate metabolism, Metabolic pathways, Microbial metabolism in diverse environments | Q4V1F5 | 1461 | 0 |
| 8I - AII09783 | *iolH*  peg.6574 | Chromosome | 5-deoxy-glucuronate isomerase (EC 5.3.1.-) | 3.3 | Iinositol catabolic process, 5-deoxy-D-glucuronate isomerase activity, glucuronate isomerase activity | Metabolic pathways, Inositol phosphate metabolism, Microbial metabolism in diverse environments | Q5KYR2 | 453 | 1.51E-53 |
| AII09784 | *iolJ*  peg.6575 | Chromosome | 5-keto-2-deoxy-D-gluconate-6 phosphate aldolase [form 2] (EC 4.1.2.29) | 3.3 | Cytoplasm, cytosol, Golgi apparatus, nuclear speck, nuclear membrane, trans-Golgi network membrane, AP-4 adaptor complex, coated vesicle membrane, extrinsic component of organelle membrane | - | Q3U3N6 | 84 | 0.212434 |
| AII09785 | *iolC*  peg.6576 | Chromosome | 5-keto-2-deoxygluconokinase (EC 2.7.1.92) | 3.3 | ATP binding, inositol catabolic process, 5-dehydro-2-deoxygluconokinase activity | Metabolic pathways, Microbial metabolism in diverse environments, Inositol phosphate | A4IPB3 | 479 | 2.56E-56 |
| AII06627 | *iolA*  peg.3111 | Chromosome | Methylmalonate-semialdehyde dehydrogenase [inositol] (EC 1.2.1.27) | -4.1 | Malonate-semialdehyde dehydrogenase (acetylating) activity, methylmalonate-semialdehyde dehydrogenase (acylating) activity, beta-alanine biosynthetic process, thymine catabolic process, valine catabolic process, valine metabolic process | Valine, leucine and isoleucine degradation, beta-Alanine metabolism, Inositol phosphate metabolism, Propanoate metabolism, Metabolic pathways, Carbon metabolism | Q9I702 | 1352 | 0 |
| TCA cycle | | | | | | | | | |
| AII07721 | *glnP*  peg.4295 | Chromosome | Glutamate permease | -2.4 | Plasma membrane, amino acid transport, transmembrane transporter activity, ATP-binding cassette (ABC) transporter complex | ABC transporters | O34606 | 295 | 1.07451E-31 |
| WP_037243137 | *artQ*  peg.4296 | Chromosome | Glutamate permease | -2.4 | Plasma membrane, amino acid transport, transmembrane transporter activity, ATP-binding cassette (ABC) transporter complex | ABC transporters | P54536 | 163 | 3.96663E-12 |
| AII07722.1 | *gluA* peg.4297 | Chromosome | Glutamate transport ATP-binding protein gluA | -2.5 | ATP binding, plasma membrane, ATPase activity, ATPase-coupled amino acid transmembrane transporter activity | ABC transporters | P48243 | 865 | 4.2148E-117 |
| AII06264 | *dctA*  peg.2727 | Chromosome | Aerobic C4-dicarboxylate transporter for fumarate, L-malate, D-malate, succunate | -2.2 | Plasma membrane, integral component of membrane, symporter activity, dicarboxylic acid transport | Two-component system | Q1J1H5 | 1041 | 5.224E-137 |
| AII04431 | *tctB*  peg.729 | Chromosome | TctB citrate transporter | -2.2 | Integral component of membrane, cholesterol metabolic process, endoplasmic reticulum membrane, endoplasmic reticulum, membrane, fatty-acyl-CoA binding, cholesterol homeostasis, cholesterol binding, cholesterol O-acyltransferase activity, sterol O-acyltransferase activity, cholesterol efflux, cholesterol esterification, cholesterol storage, low-density lipoprotein particle clearance, macrophage derived foam cell differentiation, positive regulation of amyloid precursor protein biosynthetic process, very-low-density lipoprotein particle assembly | Steroid biosynthes Cholesterol metabolismis | P35610 | 73 | 1.81033 |
| AII04432 | *tctC* peg.730 | Chromosome | TctC citrate transporter | -2.2 | - | - | O34439 | 233 | 4.11505E-21 |
| AII03701 | *aceA* peg.7759 | Chromosome | Citrate lyase beta chain (EC 4.1.3.6) | -10.9 | Magnesium ion binding, lyase activity, oxaloacetate metabolic process | Two-component system | Q9RUZ0 | 150 | 4.96369E-10 |
| AII08531 | *ppc* peg.5256 | Chromosome | Probable pyruvate carboxylase | -2.8 | Metal ion binding, ATP binding, fatty acid biosynthetic process, biotin carboxylase activity | Metabolic pathways, Biosynthesis of secondary metabolites, Microbial metabolism in diverse environments, Biosynthesis of antibiotics, Carbon metabolism, Fatty acid biosynthesis, Valine, leucine and isoleucine degradation, Pyruvate metabolism, Glyoxylate and dicarboxylate metabolism, Propanoate metabolism, Fatty acid metabolism | P46392 | 778 | 5.24203E-90 |
| AII10857 | *gnd*  peg.8702 | pPDG2 | 6-phosphogluconate dehydrogenase, decarboxylating (EC 1.1.1.44) | -5.4 | NADP binding, D-gluconate metabolic process, phosphogluconate dehydrogenase (decarboxylating) activity, pentose-phosphate shunt | Pentose phosphate pathway, Glutathione metabolism, Metabolic pathways, Biosynthesis of secondary metabolites, Microbial metabolism in diverse environments, Biosynthesis of antibiotics, Carbon metabolism | G5EBD7 | 611 | 1.36078E-76 |
| AII10884 | zwf-1  peg.8751 | pPDG2 | F420-dependent glucose-6-phosphate dehydrogenase (*fgd1*) | 4.8 | Carbohydrate metabolic process, oxidoreductase activity, acting on paired donors, with incorporation or reduction of molecular oxygen, coenzyme F420 binding, glucose-6-phosphate dehydrogenase (coenzyme F420) activity | - | Q0RVH7 | 1710 | 0 |
| AII03199 | *aceE* peg.71 | Chromosome | Pyruvate dehydrogenases E1 component | 3.95 | Glycolytic process, pyruvate dehydrogenase (acetyl-transferring) activity | Glycolysis / Gluconeogenesis, Citrate cycle (TCA cycle), Pyruvate metabolism, Metabolic pathways, Biosynthesis of secondary metabolites, Microbial metabolism in diverse environments, Biosynthesis of antibiotics, Carbon metabolism | Q59097 | 423 | 1.28E-41 |
| AII03281 | *aceE*  peg.7236 | Chromosome | Pyruvate dehydrogenases E1 component | 3.2 | Glycolytic process, pyruvate dehydrogenase (acetyl-transferring) activity | Glycolysis / Gluconeogenesis, Citrate cycle (TCA cycle), Pyruvate metabolism, Metabolic pathways, Biosynthesis of secondary metabolites, Microbial metabolism in diverse environments, Biosynthesis of antibiotics, Carbon metabolism | P45119 | 441 | 6.8E-44 |
| AII04444 | *mdh1* peg.743 | Chromosome | Malate:quinone oxidoreductase (EC 1.1.5.4) (*mqo*) | 2.7 | Tricarboxylic acid cycle, malate dehydrogenase (menaquinone) activity, malate dehydrogenase (quinone) activity | Metabolic pathways, Microbial metabolism in diverse environments, Biosynthesis of secondary metabolites, Biosynthesis of antibiotics, Carbon metabolism, Citrate cycle (TCA cycle), Pyruvate metabolism | Q0S251 | 2478 | 0 |
| WP_128642196.1 | *mdh2* peg.2088 | Chromosome | Malate dehydrogenase | 4.9 | Metal ion binding, NAD binding, malate dehydrogenase (decarboxylating) (NAD+) activity, oxaloacetate decarboxylase activity | - | P16468 | 849 | 3E-108 |
| AII04242 | *glcB* peg.531 | Chromosome | FIG00999840: hypothetical protein /2-isopropylmalate synthase activity | 2.7 | Leucine biosynthetic process, 2-isopropylmalate synthase activity | Valine, leucine and isoleucine biosynthesis, Pyruvate metabolism, Metabolic pathways, Biosynthesis of secondary metabolites, 2-Oxocarboxylic acid metabolism, Biosynthesis of amino acids | A5CRB9 | 161 | 4.05E-12 |
| AII11028 | *gabD*  peg.8974 | pPDG2 | Succinate-semialdehyde dehydrogenase [NADP+] (EC 1.2.1.16) | 4.4 | Nicotine catabolic process, succinate-semialdehyde dehydrogenase [NAD(P)+] activity | - | Q8GAI8 | 1054 | 6.6E-139 |
