# Supplementary material for: Transcriptomic Analysis of Rhodococcus opacus R7 Grown on o-Xylene by RNA-Seq
Source: Front Microbiol. 2020 Aug 12;11:1808. doi: 10.3389/fmicb.2020.01808 (PMC7434839; doi:10.3389/fmicb.2020.01808)
Supplement: Supplementary file 3 [file Table_3.docx]

**Supplementary Table S3** List of *R. opacus* R7 DEGs after RNA-seq analysis. Candidate genes for their involvement in *o*-xylene degradation pathway are listed together with their relative expression values.

| **ID NCBI** | **Gene Name** | **Location** | **Function** | **log2(fold-change)** | **GO** | **KEGG** | **Uniprot ID** | **Score** | **e-value** |
| --- | --- | --- | --- | --- | --- | --- | --- | --- | --- |
| AII11502 | *acdH* | pPDG5 | Acyl-CoA dehydrogenase-like | 5.1 | Flavin adenine dinucleotide binding, oxidoreductase activity, acting on the CH-CH group of donors, aromatic compound catabolic process, lipid catabolic process, steroid biosynthetic process, 3-hydroxy-9,10-secoandrosta-1,3,5(10)-triene-9,17-dione monooxygenase activity | Metabolic pathways, Steroid degradation, Microbial metabolism in diverse environments | Q0S811 | 470 | 1.7E-53 |
| AII11498 | *HP* | pPDG5 | Hypothetical protein | 3.6 | DNA binding, purine nucleobase metabolic process | - | O32138 | 122 | 7.44E-06 |
| AII11497 | *phaJ* | pPDG5 | Enoyl-CoA hydratase (EC:4.2.1.17) | 4.8 | Lyase activity, butyrate metabolic process | Butanoate metabolism, Carbon metabolism | P52046 | 178 | 2.18E-14 |
| WP_128638642 | *HP* | pPDG5 | Hypothetical protein | 5.6 | ATP binding, plasma membrane, integral component of membrane, phosphorelay sensor kinase activity | - | O34971 | 67 | 0.262124 |
| AII11495 | *akbT* | pPDG5 | Response regulator, two-component system | 3.8 | Cytoplasm, DNA binding, regulation of transcription, DNA-templated, phosphorelay signal transduction system | Two-component system | Q4L8Q6 | 267 | 4.27E-28 |
| AII11493 | *akbA1a* | pPDG5 | Ethylbenzene dioxygenase large subunit | 7.4 | 2 iron, 2 sulfur cluster binding, iron ion binding, toluene catabolic process, benzene 1,2-dioxygenase activity, toluene dioxygenase activity, xylene catabolic process | - | P0C618 | 777 | 6.84E-98 |
| AII11492 | *akbA2a* | pPDG5 | Ethylbenzene dioxygenase small subunit | 7.4 | Aromatic compound catabolic process, biphenyl 2,3-dioxygenase activity | Metabolic pathways, Microbial metabolism in diverse environments, Degradation of aromatic compounds , Dioxin degradation | Q53123 | 284 | 6.43E-31 |
| AII09373 | *HP* | pPDG5 | Hypothetical protein | 7.4 | Metal ion binding, 2 iron, 2 sulfur cluster binding, 3-phenylpropionate dioxygenase activity, 3-phenylpropionate catabolic process | Phenylalanine metabolism, Metabolic, Microbial metabolism in diverse environments, Degradation of aromatic compounds | Q7N4V8 | 98 | 6.36E-06 |
| AII11491 | *akbA3* | pPDG5 | Possible sterol transfer protein - ferredoxin component | 7.4 | Cytoplasm, thymidylate synthase activity, dTMP biosynthetic process, dTTP biosynthetic process, methylation | Pyrimidine metabolism, One carbon pool by folate, Metabolic pathways | Q6AFI0 | 78 | 0.140614 |
| AII11490 | *akbA4* | pPDG5 | Ferredoxin reductase | 7.4 | Flavin adenine dinucleotide binding, oxidoreductase activity | - | P43494 | 533 | 3.73E-62 |
| AII11489 | *akbB* | pPDG5 | Dihydrodiol dehydrogenase (EC1.3.1.-) | 7.4 | Aromatic compound catabolic process, cis-2,3-dihydrobiphenyl-2,3-diol dehydrogenase activity | - | P72220 | 803 | 7.5E-107 |
| AII11058 | *akbC* | pPDG2 | 1,2-Dihydroxynaphthalene dioxygenase (*meta*-cleavage dioxygenase) | 7.4 | Ferrous iron binding, aromatic compound catabolic process, biphenyl-2,3-diol 1,2-dioxygenase activity, xenobiotic catabolic process | - | P11122 | 1220 | 1.7E-169 |
| AII11057 | *aldh* | pPDG2 | Aldehydede hydrogenase (EC 1.2.1.3) | 7.4 | Aldehyde dehydrogenase (NAD+) activity, glyceraldehyde-3-phosphate dehydrogenase (NAD+) (non-phosphorylating) activity | Glycolysis / Gluconeogenesis, Fatty acid degradation, Valine, leucine and isoleucine degradation, Lysine degradation, Arginine and proline metabolism, Histidine metabolism, Tryptophan metabolism, beta-Alanine metabolism, Glycerolipid metabolism, Pyruvate metabolism, Chloroalkane and chloroalkene degradation, Metabolic pathways, Biosynthesis of secondary metabolites, Microbial metabolism in diverse environments, Biosynthesis of antibiotics | Q4L803 | 853 | 3.2E-108 |
| AII11056 | *acs* | pPDG2 | Acetyl-CoA synthetase (ADP-forming) *alpha* and *beta* chains, putative | 7.4 | Metal ion binding, ATP binding, acetate-CoA ligase (ADP-forming) activity, cofactor binding | Glycolysis / Gluconeogenesis, Pyruvate metabolism, Propanoate metabolism, Metabolic pathways, Microbial metabolism in diverse environments | O29057 | 769 | 1.01E-90 |
| AII11055 | *paaJ* | pPDG2 | Acetyl-CoA acetyltransferase (EC 2.3.1.9) -*beta*-ketoadipyl CoA thiolase (EC 2.3.1.-) | 7.4 | Fatty acid beta-oxidation, 3-oxoadipyl-CoA thiolase activity, acetyl-CoA C-acyltransferase activity, transferase activity, 3,4-dihydroxybenzoate catabolic process, cellular response to DNA damage stimulus, phenylacetate catabolic process | Phenylalanine metabolism, Metabolic pathways, Microbial metabolism in diverse environments | P0C7L2 | 1206 | 3.9E-164 |
| AII11054 | *crt* | pPDG2 | 3-Hydroxybutyryl-CoA dehydratase (EC 4.2.1.55) | 7.4 | Lyase activity, butyrate metabolic process | Butanoate metabolism, Carbon metabolism | P52046 | 548 | 1.51E-68 |
| AII11053 | *fadJ* | pPDG2 | 3-Hydroxybutyryl-CoA dehydrogenase (EC 1.1.1.157); 3-hydroxyacyl-CoA dehydrogenase (EC 1.1.1.35) | 7.4 | Cytoplasm, 3-hydroxyacyl-CoA dehydrogenase activity, NAD+ binding, butyrate metabolic process | Phenylalanine metabolism, Benzoate degradation, Butanoate metabolism, Metabolic pathways, Microbial metabolism in diverse environments, Carbon metabolism | Q0AVM2 | 518 | 1.95E-63 |
| AII11052 | *bcd* | pPDG2 | Butyryl-CoA dehydrogenase (EC 1.3.99.2) | 7.4 | Plasma membrane, flavin adenine dinucleotide binding, acyl-CoA dehydrogenase activity, oxidoreductase activity, acting on the CH-CH group of donors, with a flavin as acceptor, sporulation resulting in formation of a cellular spore | - | P45857 | 862 | 1.5E-112 |
| AII11051 | *akbD* | pPDG2 | 2-Hydroxy-6-oxo-6-phenylhexa-2,4-dienoate (HOPD) hydrolase (EC 3.7.1.-) (*meta*-cleavage hydrolase) | 7.4 | Carboxylic ester hydrolase activity, toluene catabolic process | Metabolic pathways, Microbial metabolism in diverse environments, Degradation of aromatic compounds, Xylene degradation | P23133 | 426 | 9.21E-50 |
| AII11050 | *akbE* | pPDG2 | 2-Oxo-hepta-3-ene-1,7-dioic acid hydratase (EC 4.2.-.-) | 7.4 | Metal ion binding, aromatic compound catabolic process, 2-oxo-hept-3-ene-1,7-dioate hydratase activity | - | P42270 | 762 | 1.5E-100 |
| AII11049 | *akbF* | pPDG2 | 2,4-Dihydroxyhept-2-ene-1,7-dioic acid aldolase (EC 4.1.2.-) | 7.4 | Metal ion binding, aromatic compound catabolic process, 4-hydroxy-2-oxovalerate aldolase activity | Metabolic pathways, Microbial metabolism in diverse environments, Tyrosine metabolism | O05151 | 729 | 3.79E-96 |
| AII11048 | *HP* | pPDG2 | Hypothetical protein | 11.6 | Pyridoxal phosphate binding, lyase activity, Mo-molybdopterin cofactor sulfurase activity, molybdenum cofactor sulfurtransferase activity, molybdenum ion binding, Mo-molybdopterin cofactor biosynthetic process | Folate biosynthesis | Q7SE17 | 63 | 2.30485 |
| AII11047 | *mphD* | pPDG2 | 2-Keto-4-pentenoate hydratase (EC 4.2.1.-) | 8.6 | Manganese ion binding, 3-phenylpropionate catabolic process, 2-oxopent-4-enoate hydratase activity | - | Q49KF9 | 207 | 5.78E-20 |
| AII11046 | *mphB* | pPDG2 | 3-Carboxyethylcatechol 2,3-dioxygenase (EC 1.13.11.16) | 5.5 | Ferrous iron binding, 3-phenylpropionate catabolic process, 3-carboxyethylcatechol 2,3-dioxygenase activity | Metabolic pathways, Microbial metabolism in diverse environments, Degradation of aromatic compounds, Phenylalanine metabolism | Q0SJD2 | 1403 | 0 |
| AII10989 | *matC* | pPDG2 | Dicarboxylate carrier protein | 5.0 | Extracellular region, metal ion binding, carbohydrate metabolic process, carbohydrate binding, alpha-amylase activity, alpha-amylase activity (releasing maltohexaose) | - | Q05884 | 83 | 0.634203 |
| AII10987 | *padAa* | pPDG2 | Phthalate 3,4-dioxygenase *alpha* subunit | 7.8 | 2 iron, 2 sulfur cluster binding, iron ion binding, aromatic compound catabolic process, biphenyl 2,3-dioxygenase activity | Metabolic pathways, Microbial metabolism in diverse environments, Degradation of aromatic compounds, Dioxin degradation | Q53122 | 624 | 1.9E-74 |
| AII10986 | *padAb* | pPDG2 | Phthalate 3,4-dioxygenase *beta* subunit | 7.8 | Aromatic compound catabolic process, biphenyl 2,3-dioxygenase activity | - | Q46373 | 237 | 1.53E-23 |
| AII10985 | *HP* | pPDG2 | Hypothetical protein | 7.8 |  |  |  |  |  |
| AII10984 | *padB* | pPDG2 | Dihydrodiol dehydrogenase (EC 1.3.1.56) | 7.8 | Oxidoreductase activity, acting on the CH-CH group of donors, NAD or NADP as acceptor, toluene catabolic process | Chlorocyclohexane and chlorobenzene degradation, Benzoate degradation, Toluene degradation, Polycyclic aromatic hydrocarbon degradation, Styrene degradation, Metabolic pathways, Microbial metabolism in diverse environments, Degradation of aromatic compounds | P13859 | 426 | 6.33E-50 |
| AII10983 | *padAc* | pPDG2 | Hypothetical protein | 7.8 | Iron ion binding, 3 iron, 4 sulfur cluster binding, electron transfer activity | - | P26910 | 87 | 0.000306 |
| AII10982 | *padAd* | pPDG2 | Ferredoxin reductase | 7.8 | Flavin adenine dinucleotide binding, oxidoreductase activity | - | P43494 | 566 | 3.82E-67 |
| AII10981 | *padC* | pPDG2 | COG1720: Uncharacterized conserved protein - 3,4-dihydroxyphthalate decarboxylase | 7.8 | Cytoplasm, aminopeptidase activity, manganese ion binding, metalloexopeptidase activity | Glutathione metabolism, Metabolic pathways | A0PTP9 | 79 | 0.682892 |
| AII10980 | *aldh* | pPDG2 | Aldehyde dehydrogenase (EC 1.2.1.3) | 7.8 | Aromatic compound catabolic process, oxidoreductase activity, acting on the aldehyde or oxo group of donors, NAD or NADP as acceptor | - | Q79EM7 | 1662 | 0 |
| AII09802 | *pcaG* | Chromosome | Protocatechuate 3,4-dioxygenase *beta* chain (EC 1.13.11.3) | 2.7 | 3,4-dihydroxybenzoate catabolic process, ferric iron binding, beta-ketoadipate pathway, protocatechuate 3,4-dioxygenase activity | - | P15110 | 602 | 1.72E-77 |
| AII09801 | *pcaH* | Chromosome | Protocatechuate 3,4-dioxygenase *alpha* chain (EC 1.13.11.3) | 2.7 | Ferric iron binding, beta-ketoadipate pathway, protocatechuate 3,4-dioxygenase activity | Benzoate degradation, Metabolic pathways, Microbial metabolism in diverse environments, Degradation of aromatic compounds, Polycyclic aromatic hydrocarbon degradation | P20371 | 307 | 1.01E-33 |
| AII09800 | *pcaB* | Chromosome | 3-Carboxy-*cis*,*cis*-muconate cycloisomerase (EC 5.5.1.2) | 2.7 | 3,4-dihydroxybenzoate catabolic process, beta-ketoadipate pathway, 3-carboxy-cis,cis-muconate cycloisomerase activity, 3-carboxymuconate cycloisomerase type II activity | Benzoate degradation, Metabolic pathways, Microbial metabolism in diverse environments, Degradation of aromatic compounds | Q59092 | 534 | 1.11E-61 |
| AII09799 | *pcaL* | Chromosome | 4-Carboxymuconolactone decarboxylase (EC 4.1.1.44) | 2.7 | beta-ketoadipate pathway, 3-oxoadipate enol-lactonase activity | Benzoate degradation, Metabolic pathways, Microbial metabolism in diverse environments, Degradation of aromatic compounds | P00632 | 501 | 1.57E-59 |
| AII09798 | *pcaR* | Chromosome | Pca regulon regulatory protein PcaR | 2.7 | DNA binding, aromatic compound catabolic process, positive regulation of transcription, DNA-templated, 3,4-dihydroxybenzoate metabolic process | - | Q52154 | 462 | 3.21E-55 |
| AII09797 | *pcaF* | Chromosome | Acetyl-CoA acetyltransferase (EC 2.3.1.9) | 2.7 | Acetyl-CoA C-acetyltransferase activity | Pyruvate metabolism, Metabolic pathways, Biosynthesis of secondary metabolites, Microbial metabolism in diverse environments, Biosynthesis of antibiotics, Carbon metabolism, Two-component system, Fatty acid degradation, Synthesis and degradation of ketone bodies, Valine, leucine and isoleucine degradation, Lysine degradation, Benzoate degradation, Tryptophan metabolism, Glyoxylate and dicarboxylate metabolism, Propanoate metabolism, Butanoate metabolism, Terpenoid backbone biosynthesis, Fatty acid metabolism | Q0KBP1 | 847 | 1.2E-109 |
| AII08802 | *benA* | Chromosome | Benzoate 1,2-dioxygenase *alpha* subunit (EC 1.14.12.10) | 4.0 | 2 iron, 2 sulfur cluster binding, iron ion binding, benzoate catabolic process via hydroxylation, benzoate 1,2-dioxygenase activity | Benzoate degradation, Metabolic pathways, Microbial metabolism in diverse environments, Degradation of aromatic compounds, Fluorobenzoate degradation, Xylene degradation | P07769 | 1627 | 0 |
| AII08801 | *benB* | Chromosome | Benzoate 1,2-dioxygenase *beta* subunit (EC 1.14.12.10) | 4.0 | Benzoate catabolic process via hydroxylation, benzoate 1,2-dioxygenase activity | Benzoate degradation, Metabolic pathways, Microbial metabolism in diverse environments, Degradation of aromatic compounds, Fluorobenzoate degradation, Xylene degradation | P07770 | 542 | 2.03E-70 |
| AII08800 | *benC* | Chromosome | Benzoate dioxygenase, ferredoxin reductase component (EC 1.3.1.25) | 4.0 | Metal ion binding, 2 iron, 2 sulfur cluster binding, aromatic compound catabolic process, electron transfer activity, ferredoxin-NAD+ reductase activity | - | P23101 | 962 | 2.9E-126 |
| AII08799 | *benD* | Chromosome | 1,2-Dihydroxycyclohexa-3,5-diene-1-carboxylate dehydrogenase (EC 1.3.1.25) | 4.0 | Benzoate catabolic process via hydroxylation, 1,6-dihydroxycyclohexa-2,4-diene-1-carboxylate dehydrogenase activity | - | P23102 | 839 | 1.5E-112 |
| AII08798 | *benK* | Chromosome | Benzoate MFS transporter BenK | 4.0 | Plasma membrane, integral component of membrane, transmembrane transporter activity | - | O30513 | 381 | 1.59E-39 |
| AII08684 | *fmoB* | Chromosome | Nitrilotriacetate monooxygenase component B (EC 1.14.13.-) | 9.3 | Aromatic compound catabolic process, lipid catabolic process, FMN binding, steroid biosynthetic process, flavin reductase (NADH) activity | Metabolic pathways, Steroid degradation | Q0S808 | 380 | 2.18E-45 |
| AII08680 | *bnzC* | Chromosome | 2,3-Dihydroxybiphenyl 1,2-dioxygenase (EC 1.13.11.39) | 7.2 | 3,4-dihydroxy-9,10-secoandrosta-1,3,5(10)-triene-9,17-dione 4,5-dioxygenase activity, ferrous iron binding, oxidoreductase activity, acting on single donors with incorporation of molecular oxygen, incorporation of two atoms of oxygen, aromatic compound catabolic process, cholesterol metabolic process, lipid catabolic process, response to cholesterol | Metabolic pathways, Steroid degradation, Microbial metabolism in diverse environments, Degradation of aromatic compounds | Q9KWQ5 | 702 | 1.12E-90 |
| AII08679 | *acdH* | Chromosome | Hydroxylase | 7.2 | Flavin adenine dinucleotide binding, oxidoreductase activity, acting on the CH-CH group of donors, pigment binding | - | O69349 | 1482 | 0 |
| AII08678 | *kshA* | Chromosome | Terminal oxygenase KshA | 7.2 | 2 iron, 2 sulfur cluster binding, 3-ketosteroid 9-alpha-monooxygenase activity, iron ion binding, cholesterol catabolic process | - | F1CMY8 | 1621 | 0 |
| AII05696 | *catB* | Chromosome | Muconate cycloisomerase (EC 5.5.1.1) | 4.9 | Aromatic compound catabolic process, manganese ion binding, chloromuconate cycloisomerase activity, muconate cycloisomerase activity, cellular amino acid catabolic process | - | P95608 | 820 | 2E-106 |
| WP_128639625 | *catA* | Chromosome | Catechol 1,2-dioxygenase (EC 1.13.11.1) | 4.9 | Aromatic compound catabolic process, ferric iron binding, catechol 1,2-dioxygenase activity, chlorocatechol 1,2-dioxygenase activity, catechol-containing compound metabolic | - | P95607 | 501 | 2.84E-61 |
| AII07936 | *czcO1* | Chromosome | Cyclohexanone monooxygenase (EC 1.14.13.22) | 3.5 | NADP binding, flavin adenine dinucleotide binding, N,N-dimethylaniline monooxygenase activity | ~~-~~ | A7HU16 | 1471 | 0 |
| AII05694 | *czcO6* | Chromosome | Cyclohexanone monooxygenase (EC 1.14.13.22) | 4.9 | Phenylacetone monooxygenase activity | - | Q47PU3 | 1484 | 0 |
| AII07703 | *czcO7* | Chromosome | Cyclohexanone monooxygenase (EC 1.14.13.22) | 6.3 | Phenylacetone monooxygenase activity | - | Q47PU3 | 1435 | 0 |
| AII08179 | *luxA2* | Chromosome | Alkanal monooxygenase alpha chain (EC 1.14.14.3) | 3.3 | Alkanal monooxygenase (FMN-linked) activity, bioluminescence | - | P19907 | 287 | 9.31E-28 |
| AII08186 | *rutA* | Chromosome | Predicted monooxygenase RutA in novel pyrimidine catabolism pathway | 2.7 | Monooxygenase activity, oxidoreductase activity, acting on paired donors, with incorporation or reduction of molecular oxygen, nitrogen utilization, uracil catabolic process | Pyrimidine metabolism, Metabolic pathways | A5EB33 | 718 | 2.37E-91 |
| AII06695 | *fmoB* | Chromosome | FIG00995544: hypothetical protein - nitriloacetate monooxygenase family | 2.4 | Monooxygenase activity, oxidoreductase activity, acting on paired donors, with incorporation or reduction of molecular oxygen, cysteine biosynthetic process |  | P54950 | 85 | 0.067655 |
| AII03496 | *prmD* | Chromosome | Methane monooxygenase regulatory protein B | -6.3 | Monooxygenase activity, cellular aromatic compound metabolic process | Propanoate metabolism, Metabolic pathways | Q0SJK6 | 583 | 1.984E-78 |
| AII03497 | *prmB* | Chromosome | Methane monooxygenase component A *beta* chain (EC 1.14.13.25) | -6.3 | Cellular aromatic compound metabolic process, oxidoreductase activity, acting on paired donors, with incorporation or reduction of molecular oxygen, NAD(P)H as one donor, and incorporation of one atom of oxygen | Propanoate metabolism, Metabolic pathways | Q0SJK7 | 1856 | 0 |
| AII03498 | *prmC* | Chromosome | Methane monooxygenase component C (EC 1.14.13.25) | -6.8 | Metal ion binding, 2 iron, 2 sulfur cluster binding, electron transfer activity | Propanoate metabolism, Metabolic pathways | Q0SJK8 | 1728 | 0 |
| AII03499 | *prmA* | Chromosome | Methane monooxygenase component A *alpha* chain (EC 1.14.13.25) | -7.0 | Metal ion binding, monooxygenase activity, cellular aromatic compound metabolic process | Propanoate metabolism, Metabolic pathways | Q0SJK9 | 2902 | 0 |
